# Effect of carbon fiber reinforcement on dimensional variations of 3D printed polyamide-6 composites: A simulation study

**DOI:** 10.55730/1300-0527.3513

**Published:** 2022-10-08

**Authors:** Ans AL RASHID, Hamid IKRAM, Muammer KOÇ

**Affiliations:** Division of Sustainable Development, College of Science and Engineering, Hamad Bin Khalifa University, Qatar Foundation, Doha, Qatar

**Keywords:** 3D Printing, thermoplastic composite, fused filament fabrication, warpage, digimat, residual stresses

## Abstract

Due to material design and fabrication flexibility, additive manufacturing (AM) or 3D printing (3DP) processes and polymer composites have paved their way into several industrial sectors. The quality of 3D printed polymer composites is highly dependent on the reinforcement content of polymers and 3DP process parameters. Several experimental studies are performed to optimize the reinforcement contents and process parameters; however, exploring the numerical modeling and simulation techniques is vital to lower the research and development costs. In the study, the numerical simulations for the 3DP process were performed using Digimat^®^ software for carbon fiber-reinforced polyamide-6 (PA6) composites fabricated via the fused filament fabrication (FFF) process to evaluate the effect of reinforcement content on deflections, warpages, and process-induced residual stresses. The FFF process simulations were performed to fabricate tensile testing coupons with pure PA6 and 10%–28% CF-reinforced PA6 composites. A significant impact of CF-reinforcement was observed on the deflections, warpages, and residual stresses. The maximum displacement of 4.518 mm and critical warpage of 3.012 was observed for pure PA6 material. However, with the addition of CF reinforcement, a maximum deflection of 3.369 mm and critical warpage of 2.246 was achieved for PA6 reinforced with 28% CF (PA6-CF28). The improved 3D printed specimen quality was acquired at the cost of increased residual stresses of 14.53 MPa compared to 11.75 MPa in pure PA6 specimen. The CF reinforcement significantly improved the 3DP manufacturing performance of PA6/CF composites, reducing deflections and warpages.

## 1. Introduction

Currently, researchers have improved the properties of thermoplastics by merging them with reinforcing materials [1–3]. In this regard, carbon fiber-reinforced (CFR) polymer composites are rapidly growing in structural applications like airplane parts, automotive, and other manufacturing sectors owing to their enhanced performance in terms of mechanical properties, reliability, and cost-effectiveness [4–6]. The improvement in properties is strongly influenced by the content and the length of the fibers with their orientation inside the polymer network. The high content of fiber reinforcement in the polymers can cause agglomeration, resulting in voids and limiting the fabrication process [1]. The length of the fiber is responsible for the efficient transfer of load inside the matrix network, which is characterized by a critical limit, having a diverse effect on the mechanical properties of the printed part [7]. Nevin et al. [8] evaluated the effect of fiber orientation with different fiber lengths and revealed that the distribution pattern was the same regardless of the fiber length. However, the carbon fiber content increased the tensile strength and young modulus while compromising the strain at break.

Efforts to evaluate the improvements that can be brought in the mechanical behavior of polymers, especially polyamide-6 (PA6), with the addition of fibers have been made by numerous researchers [9–11]. There are many concerns regarding the adhesion between the interfacial layers of CFR polymers due to the inert behavior of carbon fibers that develop deflections or warpages in the structure. Overall, the effect of carbon fibers on the warpage and the mechanical behavior of the polymers is complicated, and the accurate operating function is not clear quantitatively [12]. Therefore, it is considered the crucial parameter that affects the tribological parameters of the CFR structures [13].

The manufacturing of such polymers and composites is accomplished through different techniques like injection molding [14] and fused filament fabrication (FFF) [15–18]. Injection molding proceeds with the uneven distribution of the fibers through distortion resulting in a random orientation. However, the FFF technique is adopted to print 3D composite parts, which is a cost-efficient, mouldless and straightforward process requiring the modeling of the printed part [19]. Hu et al. [20] 3D printed the continuous carbon fiber-reinforced polylactic acid (PLA) samples using the prepreg filament method, which showed 61.7% higher flexural strength than the simple FDM method. Frank et al. [21] 3D printed the samples using a Markforged 3D printer for tensile testing to validate the literature for these CFR thermoplastics. Subsequently, different manufacturing techniques are utilized in the literature to evaluate the development of CFR polymer composites [22–26].

Based on the above discussion, it is evident that sufficient experimental investigation on carbon fiber-reinforced composites are accomplished; however, it is vital to explore the numerical modeling and simulation techniques to lower the research and development costs [27–30]. Therefore, this paper presents the numerical analysis of short CFR PA6 composites and their effect on the thermomechanical properties of the material. In the study, we performed the process simulations using Digimat^®^ software for CF-reinforced PA6 composites fabricated via the FFF process to evaluate the effect of reinforcement content on deflections, warpages, and process-induced residual stresses. The FFF process simulations were performed for tensile testing coupons with pure PA6 and 10%–28% CF-reinforced PA6 composites.

## 2. 3DP process simulations

Fused filament fabrication (FFF) process simulations were performed using Digimat^®^ software. A 3D CAD model for a tensile testing coupon was designed using Solidworks^®^, which was imported to slicer software (Eiger^®^) to define the toolpath information required for the process simulation. Digimat-AM^®^ module was used in this study specially designed for the 3DP process simulations. In the first step, type of manufacturing process (FFF), printer specifications (320 (X), 132 (Y), 154 (Z)), type of analysis (inherent strain), specimen geometry (tensile testing coupons according to D638) and material properties (obtained from Digimat-MX^®^ module database) are defined. In the next step, manufacturing steps (material deposition, cooling, and support removal), maximum refined element size (58.46 mm), specimen positioning (w.r.t to the print bed), toolpath information (obtained from slicer software), and 3DP process parameters (printing temperature, print speed, bead width, and chamber temperature) need to be specified. The FFF process parameters are also reported in Table 1. Finally, a mesh size of 0.5 mm was selected using reduced element integration, and a job was submitted for analysis. The same procedure was adopted for all specimens, varying the CF-reinforcement content from 0% to 28%.

## 3. Results and discussions

**Figure 1:**
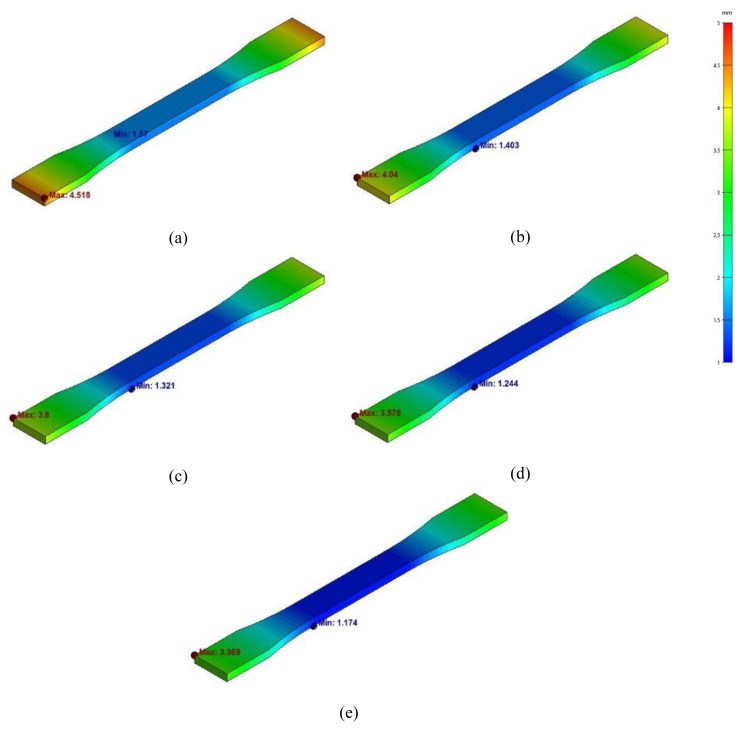
Displacement fields for (a) PA6, (b) PA6-CF10, (c) PA6-CF16, (d) PA6-CF22, (e) PA6-CF28.

**Figure 2:**
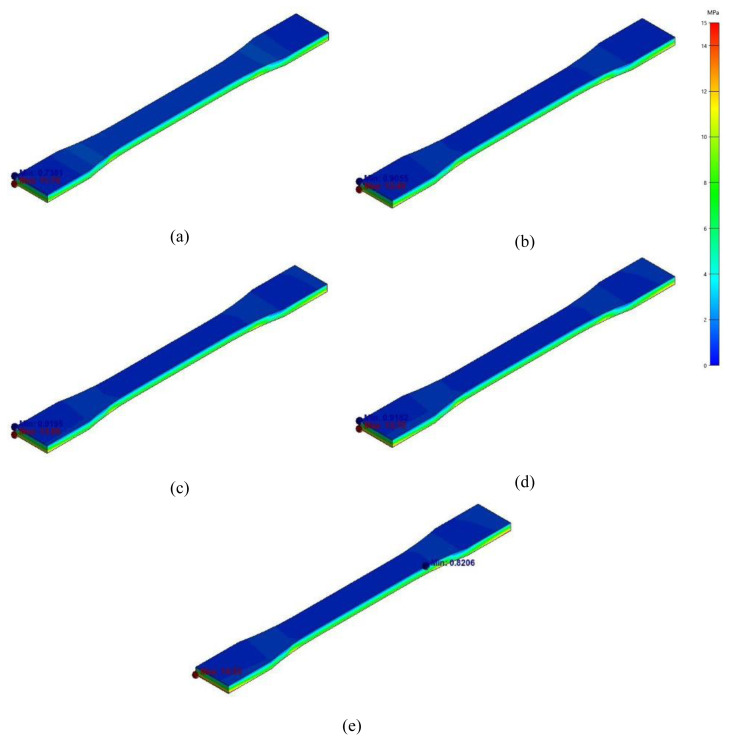
Stress fields for (a) PA6, (b) PA6-CF10, (c) PA6-CF16, (d) PA6-CF22, (e) PA6-CF28.

**Figure 3:**
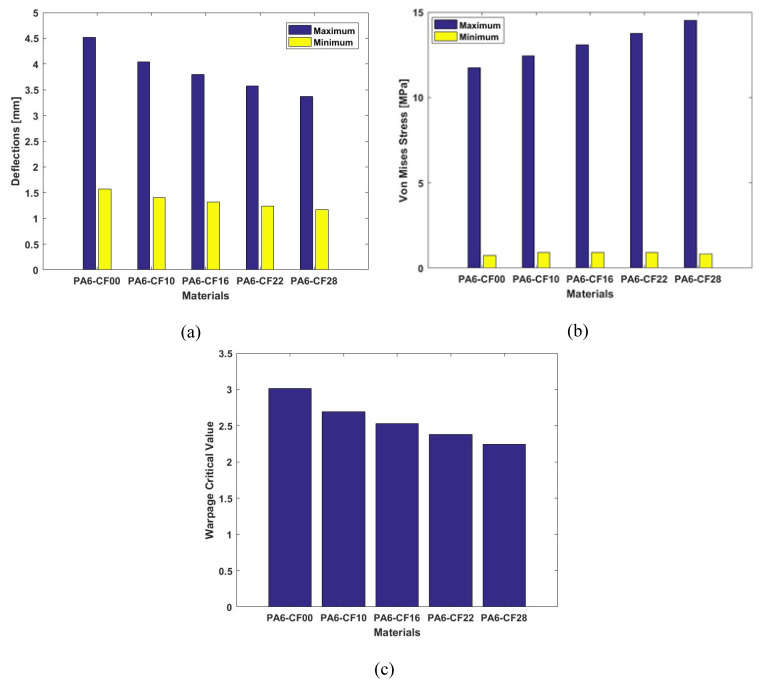
Effect of carbon fiber reinforcement on (a) deflections (b) von mises stress (c) warpage of 3D printed PA6 composites.

The deflection and stress fields are obtained after successful process simulations for all the specimens, as reported in Figures 1–2. The displacement and stress fields are plotted against the same scale of 1–5 mm and 0–15 MPa, respectively, for visual comparison of deflections, and von mises stress between different materials under consideration. The maximum and minimum deflections, von mises stresses, and warpages are presented in Figure 3. The maximum deflection for the PA6 sample was observed to be 4.518 mm from the designed specimen. The maximum deflections significantly drop with the CF-reinforcement to the PA6 polymer. The maximum deflections for 10%, 16%, 22% and 28% CF are 4.04 mm, 3.8 mm, 3.576 mm and 3.369 mm, respectively.

The warpage is a dimensionless factor that accounts for overall deformation in the 3D printed parts. The warpage critical values are 3.012, 2.693, 2.533, 2.383 and 2.246 for pure PA, PA6-CF10, PA6-CF16, PA6-CF22, and PA6-CF28, respectively.

The CF reinforcement hinders the shrinkage of material during the cooling phase, and therefore residual stresses are accumulated within the specimens. The highest value of von mises residual stresses is observed for PA6-CF28 as this specimen revealed the lowest dimensional deviations from the targeted values. The maximum von mises stress for pure PA6, PA6-CF10, PA6-CF16, PA6-CF22, and PA6-CF28 are 11.75 MPa, 12.45 MPa, 13.08 MPa, 13.75 MPa, and 14.53 MPa, respectively.

Concluding the above discussion, the deflection values drop with increased CF-reinforcement content; however, the residual stresses increase. The warpages values are also associated with the deflections and also observed to be reduced with increasing CF content. A summary of the results is also reported in Table 2.

## 4. Conclusions

In the study, numerical simulation software was used to evaluate the effect of reinforcement content on deflections, warpages, and process-induced residual stresses in the FFF process. A significant impact of CF-reinforcement was observed on the deflections, warpages, and residual stresses. The CF reinforcement significantly improved the 3DP manufacturing performance of PA6/CF composites, reducing deflections and warpages. The deflection values drop with increased CF-reinforcement content; however, the residual stresses increase. The CF reinforcement hinders the shrinkage of material during the cooling phase, and therefore residual stresses are accumulated within the specimens. The warpages values are also associated with the deflections and also observed to be reduced with increasing CF content. Future studies will consider comparing experimentally observed dimensional variations of FFF fabricated specimens to validate the numerical simulation results. In addition, the effect of process parameters can be monitored via process simulations to optimize print quality.

## Figures and Tables

**Table 1:** Digimat-AM^®^ process parameters settings.

Chamber temperature	100 °C
Extrusion temperature	250 °C
Build plate temperature	115 °C
Bead width	0.4
Convection coefficient	0.015mW/mm^2^.°C
Final/room temperature	23 °C
Printing speed	60 mm/s

**Table 2:** Summary of thermomechanical characteristics for CF-reinforced PA6 composites.

Specimen		1	2	3	4	5
**Polymer**		PA6	PA6	PA6	PA6	PA6
**CF Reinforcement**		-	10%	16%	22%	28%
**Deflection (mm)**	**Max**	4.518	4.040	3.80	3.576	3.369
	**Min**	1.570	1.403	1.321	1.244	1.174
**Stress (MPa)**	**Max**	11.75	12.45	13.08	13.75	14.53
	**Min**	0.738	0.906	0.919	0.918	0.821
**Warpage**		3.012	2.693	2.533	2.383	2.246
